# Conformational control over π-conjugated electron pairing in 1D organic polymers†

**DOI:** 10.1039/d1ra03187b

**Published:** 2021-06-08

**Authors:** Isaac Alcón, Jingjing Shao, Jean Christophe Tremblay, Beate Paulus

**Affiliations:** Institut für Chemie und Biochemie, Physikalische und Theoretische Chemie, Freie Universität Berlin Arnimallee 22 14195 Berlin Germany ialcon8@gmail.com; CNRS-Université de Lorraine, LPCT 57070 Metz France

## Abstract

During the past decades π-conjugated bi-radicals have attracted increasing attention, due to the existence of two close-in-energy resonant electronic configurations with very distinct characteristics: the open-shell bi-radical and the closed-shell quinoidal. The chemical design of the bi-radical structure has been shown to be very effective to shift the balance towards one, or the other, electronic distribution. Some reports have experimentally studied the analogous 1D oligomers and polymers, however, only the open-shell multi-radical configuration has been detected, and it is yet not very clear which structural and chemical parameters are relevant in such extended systems. In this work, *via* first principles quantum chemical simulations, we study a series of π-conjugated 1D polymers based on triarylmethyl radicals with different chemical functionalization. We find that dihedral angles of the aryl rings connecting the radical centres are the key conformational parameter determining the electronic balance. This provides a simple recipe to use chemical functionalization of aryl rings as a tool to shift the system towards either the electron paired or unpaired configurations. Additionally, we find such conformational control is also effective under the effect of thermal fluctuations, which highlights its potential technological applicability.

## Abstract 2

Chemically designed conformational changes are shown to act as effective tools to induce electron pairing in otherwise multiradical 1D organic polymers.

## Graphical abstract

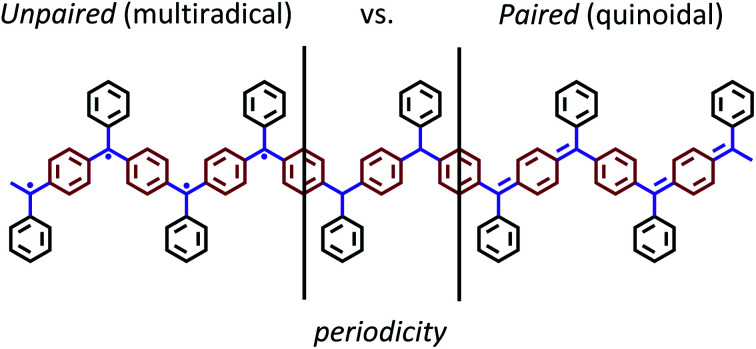

## Introduction

**Fig. 1:**
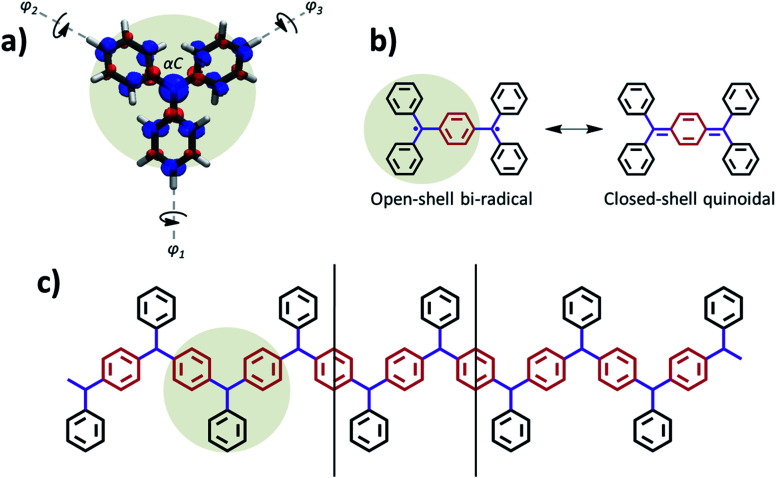
(a) Basic structure of TAM radicals, where the unpaired electron mainly resides on the central carbon atom (αC) but it also partially delocalizes into the three twisted aryl rings, as shown with the associated spin density (spin-up: blue; spin-down: red; iso-surface value: 0.005 e bohr^−3^) obtained from a spin-unrestricted DFT calculation using the PBE0 functional.^5,32^ (b) Lewis resonance forms associated to the two electronic solutions existing in Thiele's hydrocarbon: open-shell bi-radical (left) and closed-shell quinoidal (right). The aryl ring which accommodates electron pairing in the later is coloured in red. (c) Basic skeleton of the ring-sharing *p*-TAM 1D polymers considered in this study, with the same colouring as in (b). Vertical lines indicate the repeating unit cell along the periodic polymer direction. The monomeric TAM structure is highlighted in (a–c) to facilitate its visualization.

The first π-conjugated organic radical was reported by Moses Gomberg at the beginning of the twentieth century,^1^ known as triphenylmethyl (TPM). TPM belongs to the class of organic compounds known as triarylmethyls (TAMs),^2,3^ which are composed of a central methyl carbon atom (αC) connected to three aryl rings.^4^ The unpaired electron in TAMs is mainly located on the αC but, due to the π-conjugated nature of the system, it may delocalize through the three aryl rings (see spin density in Fig. 1a).^5^ TAMs are kinetically stable due to the steric protection that the three aryl rings provide to the radical centre. During the second half of the twentieth century it was shown that such kinetic stability could be enormously increased by functionalizing the three aryl rings with chlorine atoms.^6^ Such increased stability allowed utilizing TAMs as stable building blocks for various types of materials and molecular devices,^7^ exploiting their unique physicochemical properties associated with their π-conjugated unpaired electron. For instance, in the last few decades TAMs have been utilized to construct magnetic plastics,^8,9^ magnetic metal–organic frameworks,^10,11^ electrochemical optical switches^12–14^ and, more recently, electronic^15,16^ and spintronic^17^ molecular devices.

Due to their fully π-conjugated structure, the properties of TAM-based dimers and trimers depend on the way the radical αC centres are connected. Meta-connections lead to localized unpaired electrons^18^ with ferromagnetically coupled spins (*i.e.* triplet states).^3^ For this reason *meta*-connected TAM 1D polymers^19^ gathered great attention in the past to realize organic magnets.^8,9^ On the other hand, *para*-connected TAM dimers, such as Thiele's hydrocarbon (Fig. 1b),^20^ belong to the family known as Kekulé bi-radicals, which show a bi-stable electronic structure composed of two energetically close, but physicochemically distinct, electronic resonant configurations: namely the open-shell bi-radical (OS) and the closed-shell quinoidal (CS, see Fig. 1b). Thanks to the very distinct magnetic,^21^ optical^22,23^ and structural^24^ properties between these two electronic states, Kekulé bi-radicals have attracted great attention in the last decades in the fields of molecular electronics^25–28^ and magnetism.^9^ To the best of our knowledge, para-connected TAM polymers were only experimentally studied several decades ago in one single study,^29^ although recently the electronic structure of other types of Kekulé 1D oligomers has been assessed by different in-solution and on-surface spectroscopies.^30,31^ In all these cases a complete, or partial, multi-radical character was found, which is in agreement with the higher degree of aromatization in the open-shell configuration, making it generally more stable than the fully paired quinoidal distribution.^21^ Therefore, it is not very clear how the quinoidal configuration could be stabilized in such type of *para*-connected TAM polymers, or what is the effect of chemical functionalization on the resulting electronic configuration, as opposed to TAM bi-radicals where this has been extensively studied in the past.^9^

In this work, we study four *para*-connected TAM 1D-polymers (*p*-TAM polymers) with diverse chemical functionalization using periodic density functional theory (DFT) calculations. We separately study the open-shell multi-radical and closed-shell quinoidal electronic solutions, which allows us to assess their relative energetic stability and their effect on the structural conformation of the polymers. We find that dihedral angles of aryl rings connecting the radical αC centres along the polymeric direction are the key structural parameters determining the balance between the two electronic states. *Ab initio* molecular dynamics simulations further confirm this finding at finite temperatures, which highlights the robustness of this conformational/electronic correlation. As we demonstrate, this allows us to provide a simple general strategy to design *p*-TAM polymers with either open-shell or closed-shell character, and thus with specific electronic, magnetic and optical properties for target applications.

### Models and methods

The electronic structure of *p*-TAM polymers is characterized by means of periodic DFT, using the hybrid PBE0 functional,^32^ which was previously found to provide reliable electronic structure for TAM oligomers and 2D networks.^33–36^ The calculations are done separately for the open-shell multi-radical and closed-shell quinoidal electronic solutions. The multi-radical solution is obtained from spin-unrestricted DFT calculations setting an *anti*-parallel spin initial guess on neighbouring αC centres. The closed-shell quinoidal solution is obtained from spin-restricted DFT calculations. The atomic structure of the 1D polymers and the *a* unit cell parameter (*x*-direction) are pre-optimized using the PBE^37^ functional and a Tier-1 light numerical atom-centered orbital (NAO) basis set,^38^ as implemented in the Fritz Haber Institute *ab initio* molecular simulations package (FHI-AIMS).^39,40^ These pre-optimizations are followed by full optimizations (atomic coordinates and *a* unit cell parameter) using the PBE0 hybrid functional^32^ and the same light NAO basis set. We note that the PBE0 functional has been previously shown^36^ to properly reproduce experimentally measured magnetic coupling coefficients of a synthesized circular *p*-TAM oligomer.^41^ PBE and PBE0 optimizations are done employing a 6-1-1 and 18-1-1 Γ-centred Monkhorst Pack (MP) *k*-grid, respectively. The convergence criteria are set to 1 × 10^−5^ eV for the total energy and 1 × 10^−2^ eV Å^−1^ for the maximum force component per atom. Finally, the electronic structure is generated for the fully optimized structures using the PBE0 functional, a 36-1-1 Γ-centred MP *k*-grid, and a Tier-2 tight NAO basis set.^40,42^ All band structures and electronic (spin) density maps reported in this work are generated from these last single-point calculations, as well as other reported quantities such as total energies, atomically-partitioned αC spin populations (using the Hirshfeld method^43^), and electronic bandgaps. Atomically-partitioned spin populations (*μ*_i_) are calculated as the difference in number of electrons for spin up and spin down channels at each atom, as implemented in the FHI-AIMS code. Spin density plots are calculated similarly: *i.e.* as an electron density difference. *Ab initio* molecular dynamics simulations (AIMDS) are run at 300 K for 5 ps (1 ps equilibration + 4 ps production) using the FHI-AIMS software. These calculations are done using the Bussi–Donadio–Parrinello thermostat,^44^ the PBE0 functional, a 6-1-1 Γ-centred MP *k*-grid, and Tier-1 light NAO basis set.

## Results and discussion

### Materials design

It has been established in previous work that dihedral angles between aryl rings connecting the radical centres in Kekulé-type bi-radicals determine the preference for either the open-shell or the closed-shell electronic solutions.^23^ This also applies for TAM-based systems, as the degree of delocalization of the unpaired electron in TAMs almost entirely depends on the twist angle of aryl rings^5^ (see *φ*_1_, *φ*_2_ and *φ*_3_ in Fig. 1a). In this spirit, control over the aryl ring twist *via* chemical functionalization is a promising approach in order to design TAM-based materials with predefined electronic properties (*e.g.* magnetic susceptibility, optical absorption/emission) determined by a specific open-shell/closed-shell balance. This strategy has been recently pursued for 2D-covalent organic frameworks (2D-COFs) based on TAMs.^35^ There it has been shown that, in order to obtain a tunable electron pairing at room temperature *via* dihedral angle manipulation, the αC centres should be separated by a single aryl ring,^36^ in line with previously reported trends for TAM dimers.^9,21^ On the contrary, TAM 2D-COFs where radical centres are further away from each other, such as the recently synthesized materials,^45–47^ show a persistent multi-radical configuration that is insensitive to aryl ring flattening.^36^

**Fig. 2:**
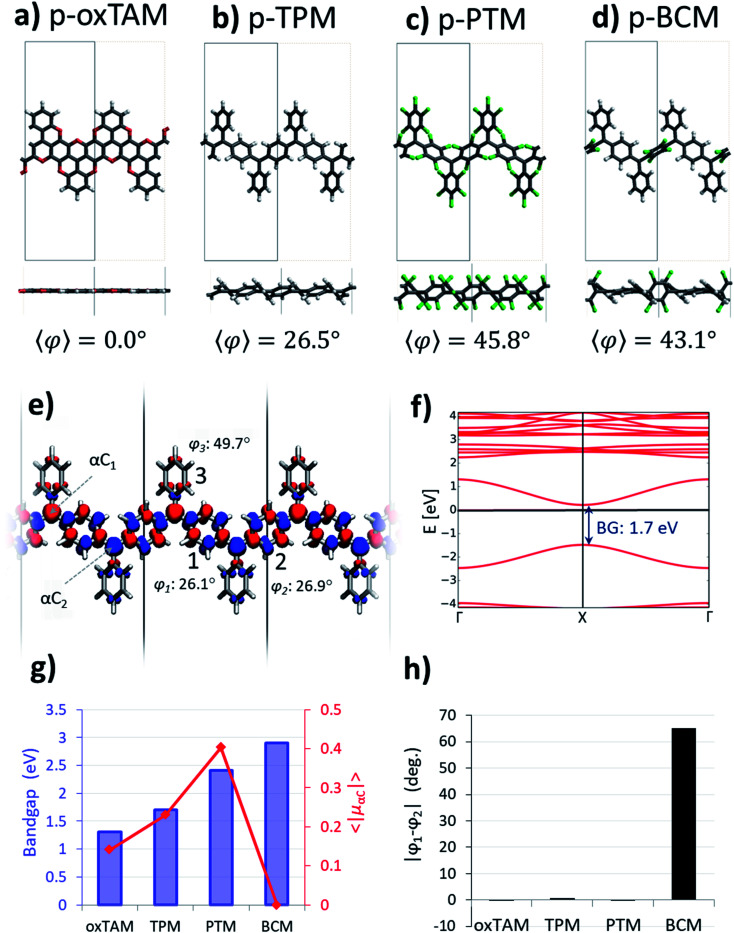
Optimized atomic periodic structures in the open-shell multi-radical solution for (a) *p*-oxTAM, (b) *p*-TPM, (c) *p*-PTM and (d) *p*-BCM polymers. Atom colour key: C – grey, H – white, O – red, Cl – green. The unit cell is repeated twice to facilitate the visualization of the periodic structure, also providing at the bottom the associated mean dihedral angle calculated as 
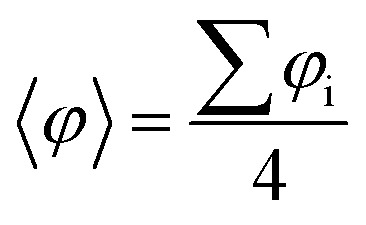
 where *φ*_i_ are the dihedral angles of each of the four aryl rings within the unit cell. (e) Spin density isosurface (spin up: blue; spin down: red; iso-surface value: 0.003 e bohr^−3^) in the *p*-TPM polymer and (f) associated spin-polarised electronic band structure (spin up: blue; spin down: red). Note that spin up and spin down bands are perfectly superimposed. The Fermi energy is marked with a black horizontal line. (g) Electronic band gap values (blue bars) and average of the absolute spin population on αCs (〈|*μ*_αC_|〉; red curve) throughout the series. (h) Dihedral angle difference between the two aryl rings connecting αC centres (1 and 2 in e) for all materials.

Based on these findings, here we consider ring-sharing *p*-TAM polymers (Fig. 1c) which may be understood as the 1D periodic analogues of Thiele's hydrocarbon (Fig. 1b). Concretely, as shown in Fig. 2a–d, we study the *para*-oxo-triarylmethyl^48^ (*p*-oxTAM), *para*-triphenylmethyl^1^ (*p*-TPM), *para*-perchlorotriarylmethyl^6^ (*p*-PTM) and *para*-biphenylchloroarylmethyl (*p*-BCM) polymers, respectively. These 1D materials are studied *via* periodic DFT calculations, using a computationally efficient minimal unit cell which captures the studied electronic interplay, while allowing for the very computationally demanding AIMDS shown below. Longer unit cells (*e.g.* ×6) do not lead to a new conformational energetic minimum, as tested for the most flexible of our considered polymers, the *p*-TPM (see Fig. S1†). This supports the validity of the minimal periodic representation utilized throughout our study. For each polymer, we characterize the two possible resonant electronic solutions existing in *para*-connected TAM systems, namely the open-shell multi-radical and the closed-shell quinoidal.

### Open-shell multiradical solution

The open-shell electronic solution in *p*-TAM polymers is obtained *via* spin unrestricted calculations and setting an antiparallel spin alignment between neighbouring αCs as initial guess. Fig. 2a–d show the optimized periodic structures for the *p*-oxTAM, *p*-TPM, *p*-PTM and *p*-BCM polymers. The chemical functionality along the series of polymers provides a wide range of aryl ring twist angles (see *y*-view in Fig. 2a–d), from fully planar (2a) to highly twisted (2c and d) conformations. Fig. 2e shows the spatial spin density distribution for the *p*-TPM polymer, revealing an antiferromagnetic spin alignment between neighbouring αC centres, typical of *para*-connected bi-radicals,^9^ as well as a strongly delocalized character. Such delocalization is more significant in the aryl rings along the polymer direction (1 and 2 in Fig. 2e) than in the peripheral ones (3). This may be explained by the more severely twisted conformation of the latter (see values for *φ*_1_, *φ*_2_ and *φ*_3_ in Fig. 2e). We note that such spin alternation is also found for the other 1D polymers where the open-shell multiradical solution is accessible (see Fig. S2†). Fig. 2f shows the band structure associated with the open-shell solution depicted in Fig. 2e. Note that spin-up and spin-down bands are perfectly superimposed. The bands associated to unpaired electrons (first occupied and unoccupied bands around the Fermi energy) have a significant band dispersion along Γ → X, which is characteristic of conductive (or delocalized) states. A direct band gap is found at the X-point for all polymers. In the case of the *p*-TPM, it takes the value of 1.7 eV. The *p*-PTM and *p*-oxTAM show a qualitatively similar band structure (see Fig. S2†). However, an increased twisting of the aryl rings along the series (see 〈*φ*〉 in Fig. 2a–d) also leads to increasing bandgaps (see blue bars in Fig. 2g) and to lower band dispersion (see Fig. S2†).

In order to quantify the open-shell character through the *p*-TAM polymers series we extract the average of the absolute spin population on the αCs (〈|*μ*_αC_|〉 = (|*μ*_αC1_| + |*μ*_αC2_|)/2). This measure is typically used to characterize the open-shell/closed-shell balance in extended TAM systems.^34–36^Fig. 2g shows 〈|*μ*_αC_|〉 for the 1D material series (red curve). On the one hand, we may see that the *p*-oxTAM, *p*-TPM and *p*-PTM show increasing 〈|*μ*_αC_|〉 values which may be explained by the increasing mean dihedral angle along the series (see 〈*φ*〉 in Fig. 2a–c). This is consistent with the expected effect of twisting the aryl rings on spin localization,^5^ and with the increasing band gaps along these materials (see blue bars in Fig. 2g). However, the *p*-BCM breaks this trend: 〈|*μ*_αC_|〉 vanishes completely in spite of its high 〈*φ*〉 equal to 43.1° (Fig. 2d). This apparently anomalous behaviour can be explained using another conformational parameter not introduced so far: the dihedral angle difference (|*φ*_1_ − *φ*_2_|) between the two aryl rings along the polymer direction (1 and 2 in Fig. 2e). Aryl rings 1 and 2 are the most relevant ones because they connect αCs along the polymer direction. They are thus the ones accommodating electron pairing when that occurs. As shown in Fig. 2h, these two aryl rings are equally twisted (|*φ*_1_ − *φ*_2_| ≈ 0) for the *p*-oxTAM, *p*-TPM and *p*-PTM. However, within the *p*-BCM |*φ*_1_ − *φ*_2_| is as high as 65°. This arises from the chlorination of ring 2, which is found highly out-of-plane (*φ*_2_ = 75.6°) due to steric hindrance, while phenyl ring 1 remains in a nearly planar conformation (*φ*_1_ = 10.6°). Such dihedral difference significantly increases the electronic coupling along ring 1, as compared to ring 2 where it almost vanishes. As a consequence, electron pairing takes place within aryl ring 1 and the open-shell multiradical solution becomes energetically unfavourable for the *p*-BCM polymer, despite biasing the electronic structure by using an antiferromagnetic initial guess in the DFT calculation.

### Closed-shell quinoidal solution

**Fig. 3:**
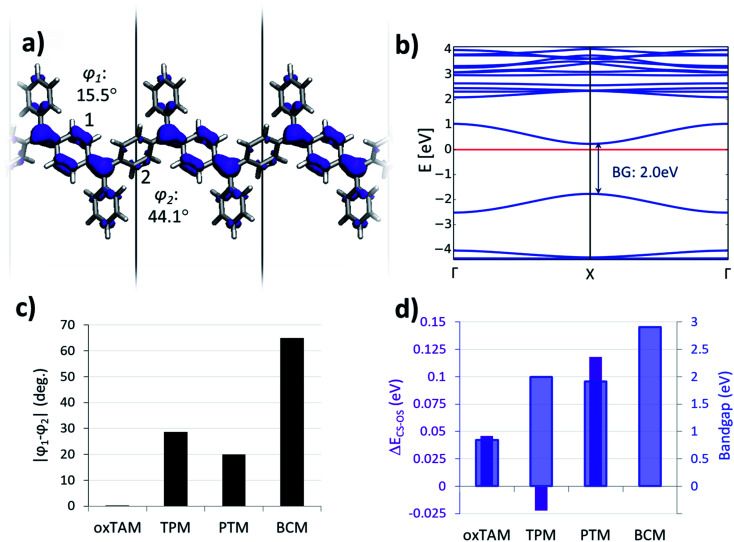
(a) Highest occupied crystal orbital density for the closed-shell quinoidal solution for the *p*-TPM polymer (purple; iso-surface value: 0.002 e bohr^−3^). (b) Associated band structure (the Fermi energy is marked with a red horizontal line). (c) Dihedral angle difference between the two aryl rings connecting αC centres (1 and 2 in a) for all materials. (d) Electronic band gap values (blue bars) and relative total energies with respect to the open-shell multi-radical electronic solution (purple bars).

In order to better understand such paired closed-shell solution along the *p*-TAM series, we re-optimize the atomic and electronic structure of each 1D material using restricted DFT, thus forcing all electrons to be paired. Fig. 3a shows the electron density of the highest occupied crystal orbital for the *p*-TPM, where the quinoidal configuration can be recognized, as sketched in Fig. 1b for Thiele's hydrocarbon. This electronic distribution is the same for all the other *p*-TAM polymers (see Fig. S3†). Localized electron pairing gives rise to a semiconductor-like band structure, characterized by a non-negligible dispersion of the two bands around the Fermi energy, separated by a finite bandgap of nearly 2 eV (Fig. 3b). Note that the band structures for the other 1D polymers are qualitatively similar (see Fig. S3†), although the associated bandgaps vary strongly (see blue bars in Fig. 3d).

As it may be seen in Fig. 3a, the quinoidal closed-shell configuration leads to the planarization of the aryl ring accommodating electron pairing (*φ*_1_ = 15.5°). Simultaneously, the neighbouring aryl ring rotates out-of-plane (*φ*_2_ = 44.1°). Such conformational changes, previously reported for the analogous 2D materials,^34^ arise from the formation of double-bonds between αC centres and their first atom neighbours within aryl ring 1. Fig. 3c shows the dihedral angle difference between the two aryl rings (|*φ*_1_ − *φ*_2_|) for all considered polymers. On the one hand, *p*-oxTAM shows no difference in the twist angle. This is consistent with its rigidly planar structure, due to the bridging oxygen atoms between adjacent aryl rings. The *p*-TPM and *p*-PTM show a |*φ*_1_ − *φ*_2_| of 30° and 20°, respectively, which highlights the significant conformational impact of electron pairing in these materials. For comparison, the corresponding open-shell solutions are found at |*φ*_1_ − *φ*_2_| ≈ 0 in both cases. The slightly higher |*φ*_1_ − *φ*_2_| for the *p*-TPM may be associated to the lower steric hindrance of phenyl rings as compared to the perchlorinated ones, leading to a higher rotational freedom.^5^ However, the *p*-BCM is, by far, the polymer showing the highest |*φ*_1_ − *φ*_2_| value (65°). As previously explained, such a high dihedral angle difference arises from the specific chemical functionalization of *p*-BCM, where aryl ring 1 is fully hydrogenated whereas aryl ring 2 is fully chlorinated. This leads to a strong out-of-plane conformation for aryl ring 2 which, in turn, gives rise to a planarization of aryl ring 1, thereby strongly promoting electron pairing in it.

Fig. 3d shows the relative energy of the closed-shell quinoidal solution with respect to the open-shell multiradical for each *p*-TAM polymer (Δ*E*_CS–OS_) and the band gap for the quinoidal configuration. Note that Δ*E*_CS–OS_ cannot be calculated for the *p*-BCM because the open-shell electronic solution could not be found. This may be regarded as a sign of high preference for the quinoidal configuration in this 1D material. With respect to the rest of the *p*-TAM polymers, the electron paired configuration is the ground state only for *p*-TPM. This arises from the low steric hindrance of phenyl rings which, in turn, allows for a facile aryl ring planarization strongly stabilizing electron pairing. In the more structurally rigid *p*-oxTAM and *p*-PTM the open-shell multi-radical solution is significantly more stable, particularly for the later one, where the conformational changes induced by quinonization, such as aryl ring flattening, are energetically very unfavourable. This, in turn, favours the less structurally constrained open-shell solution. The band gap variation along the series (blue bars in Fig. 3d) shows a similar trend as for the open-shell multi-radical solution (Fig. 2g), with the exception of the *p*-TPM which has a slightly higher gap compared to the *p*-PTM. This relative change may be understood by the higher symmetry breaking in the former caused by the larger |*φ*_1_ − *φ*_2_| change as compared to *p*-PTM (Fig. 3c), leading to a more localized electron pairing, and thus higher band gap. In the *p*-BCM this effect is even more pronounced and so it shows the highest electronic band gap of all considered materials.

### Electronic interplay at finite temperatures

**Fig. 4:**
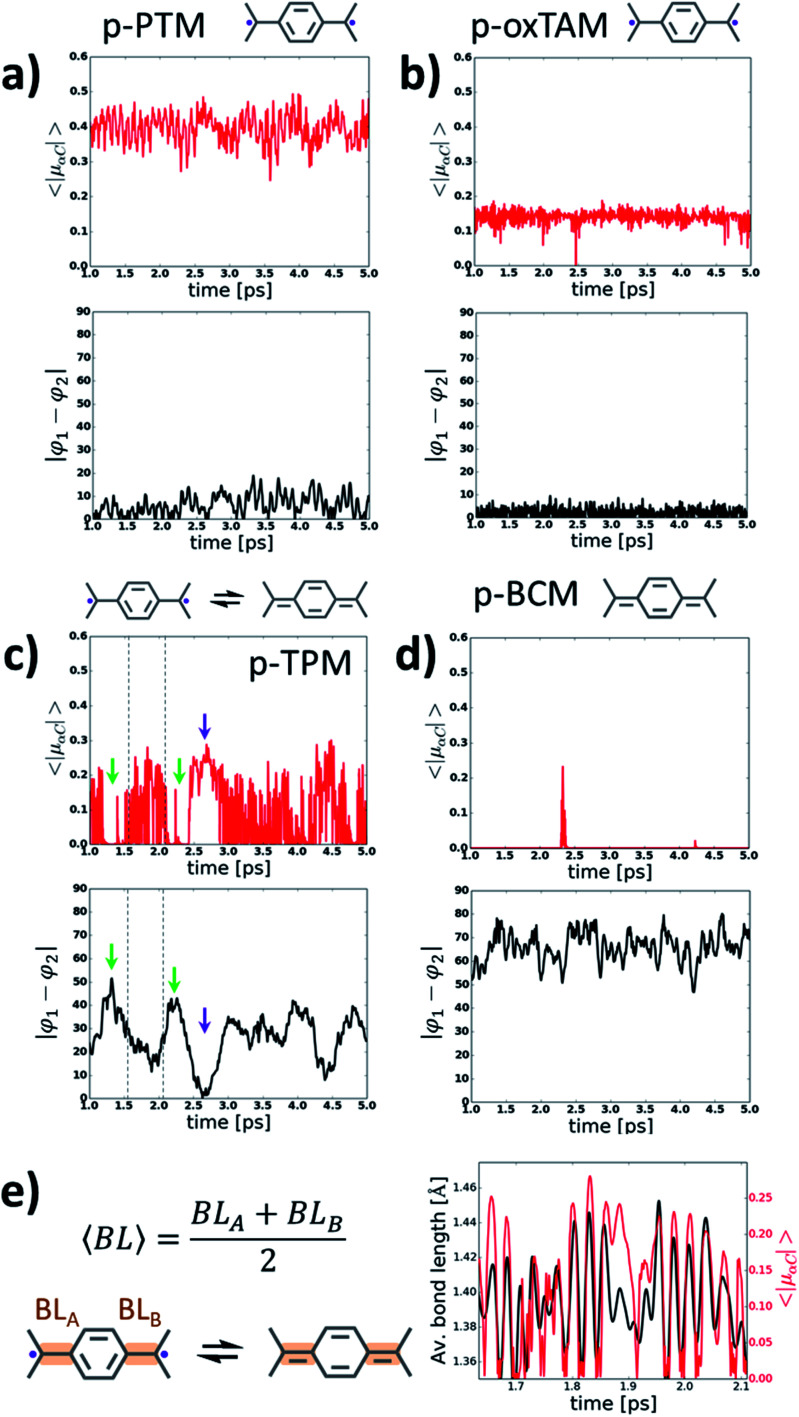
Variation of the average of the absolute spin population on αCs (〈|*μ*_αC_|〉; top panels) and dihedral angle difference between the two aryl rings connecting αCs (|*φ*_1_ − *φ*_2_|, bottom panels) during 4 ps of a molecular dynamics at 300 K for (a) *p*-PTM, (b) *p*-oxTAM, (c) *p*-TPM and (d) *p*-BCM polymers. Sketches of the predominant electronic configuration for each case are provided at the top. (e) Variation of the average αC bond length (〈BL〉) for aryl ring 1 in the *p*-TPM polymer (black curve) and 〈|*μ*_αC_|〉 (red curve) extracted from the dynamics for a short time frame (see dashed lines in c). 〈BL〉 is calculated as outlined in the left panel. Note that aryl ring 1 is the most planar aryl ring during that time frame (see *φ*_1_ in Fig. S4c†).

In order to gain more insight into the behaviour of *p*-TAM polymers at finite temperatures, we perform *ab initio* molecular dynamics simulations (AIMDS) for each system at 300 K (see Methods for details). Fig. 4 shows the variation of 〈|*μ*_αC_|〉 (top panels) and |*φ*_1_ − *φ*_2_| (bottom panels) during the dynamics for all considered *p*-TAM polymers, obtained after an initial 1 ps thermalization period. As shown above, |*φ*_1_ − *φ*_2_| is an appropriate measure to detect those structural conformations which lead to electron pairing (*i.e.* high |*φ*_1_ − *φ*_2_| values) or electron unpairing (*i.e.* low |*φ*_1_ − *φ*_2_| values). Three types of behaviour can be distinguished in Fig. 4. First, the *p*-PTM and *p*-oxTAM show an approximately constant and finite 〈|*μ*_αC_|〉 during the entire simulation, indicative of a robust and stable open-shell character at room temperature. This is in agreement with the energetic stability of such configuration in these materials at zero temperature (see Δ*E*_CS–OS_ in Fig. 3d). This, in turn, arises from the structural rigidity of both polymers, in which aryl rings 1 and 2 are almost equally twisted at all times (see |*φ*_1_ − *φ*_2_| in Fig. 4a and b). As previously explained, this conformational situation prevents electron pairing. The lower value of 〈|*μ*_αC_|〉 for the *p*-oxTAM as compared to the *p*-PTM polymer may be understood by its fully planar structure (see *φ*_1_ and *φ*_2_ in Fig. S4b†), which strongly delocalizes spin density (see Fig. S2b†). On the contrary, the highly twisted conformation of *p*-PTM leads to a significant localization of unpaired electrons on the αCs (see *φ*_1_ and *φ*_2_ in Fig. S4a†), thus increasing 〈|*μ*_αC_|〉 (Fig. 4a).

For the *p*-TPM polymer we may observe a very different behaviour (Fig. 4c), with various regimes during the dynamics depending on the values of |*φ*_1_ − *φ*_2_|. For time frames when |*φ*_1_ − *φ*_2_| is maximal there is a significant decrease of 〈|*μ*_αC_|〉 (see green arrows in Fig. 4c). Contrary, when |*φ*_1_ − *φ*_2_| is very low, a transitory open-shell configuration emerges, as detected with the corresponding rise of 〈|*μ*_αC_|〉 (see purple arrows in Fig. 4c). However, for the remaining time when the *p*-TPM polymer shows moderate |*φ*_1_ − *φ*_2_| values, one may observe rapid fluctuations of 〈|*μ*_αC_|〉 which may be ascribed to fast transitions between the paired and unpaired configurations. As shown in Fig. 4e for a selected time frame (see dashed lines in Fig. 4c), the frequency of such fast 〈|*μ*_αC_|〉 fluctuations coincides with the vibration frequency of bonds between αCs and the flattened aryl ring. Therefore, in those situations where the electronic configuration is not fully determined by aryl ring dihedral angles (*i.e.* for intermediate |*φ*_1_ − *φ*_2_| values) αC bond lengths become a relevant conformational parameter determining the pairing or unpairing of π-conjugated electrons. This particularly complex situation for the *p*-TPM polymer correlates well with the energetic proximity between the closed-shell and open-shell electronic solutions in such material (see Δ*E*_CS–OS_ in Fig. 3d).

At last, with the exception of few spurious 〈|*μ*_αC_|〉 peaks, the *p*-BCM polymer shows a vanishing 〈|*μ*_αC_|〉 along the entire AIMDS, which demonstrates the robustness of electron pairing in this material against thermal fluctuations. This is in full agreement with the results at 0 K, which also highlight the particular stability of the closed-shell quinoidal electronic solution in *p*-BCM. This result may be explained by the large and constant |*φ*_1_ − *φ*_2_| value (bottom panel in Fig. 4d) which originates from the specific chemical functionalization of the *p*-BCM polymer. Therefore, contrary to the *p*-TPM, the dihedral difference in this case provides a sufficient stabilization for the quinoidal configuration, making electron pairing highly robust. Finally, we note that the band gap of all *p*-TAM polymers fluctuates around the values found at 0 K (see Fig. S5†) and such variations are correlated with the structural fluctuations within each material (*i.e.* aryl ring rotations and bond vibrations). Overall, the results of Fig. 4 allow us to identify the different structural variables which play a role on determining the open-shell/closed-shell character of *p*-TAM polymers at finite temperatures. Additionally, they also highlight the potential of chemical functionalization for designing such 1D polymers with pre-selected open-shell (Fig. 4a and b) or closed-shell (4d) electronic configurations that remain robust at room temperature.

## Conclusions

In this work we have studied the electronic structure of *p*-oxTAM, *p*-TPM, *p*-PTM and *p*-BCM polymers, based on the respective TAM monomers,^1,6,48,49^*via* first principles DFT calculations and AIMD simulations. We find that, as for the analogous bi-radical compounds,^20,21,23^*p*-TAM polymers support two electronic solutions at similar energies with very different physicochemical properties: the open-shell multi-radical and the closed-shell quinoidal. The key structural parameter determining the balance between both electronic solutions is the dihedral angle difference between neighbouring aryl rings placed along the polymer direction (|*φ*_1_ − *φ*_2_|). Those aryl rings are, in turn, the ones connecting neighbouring radical centres (αCs), and so they play a critical role in the electron pairing mechanism. *p*-TAM polymers with a symmetric functionalization of aryl rings tend to have low |*φ*_1_ − *φ*_2_| values, thereby intrinsically favoring the open-shell electronic solution, unless the aryl rings have significant rotational freedom. In such case, about equal population of both solutions arises at room temperature (*e.g. p*-TPM polymer), where different electronic regimes are found during the dynamics depending on the |*φ*_1_ − *φ*_2_| fluctuations. For some intermediate values of |*φ*_1_ − *φ*_2_|, αCs bond vibrations become relevant, giving rise to fast open-shell/closed-shell transitions. On the contrary, *p*-TAM polymers with an asymmetric aryl ring functionalization (*e.g. p*-BCM) and, consequently, high |*φ*_1_ − *φ*_2_| values, show a particularly stable closed-shell quinoidal configuration, for which the open-shell solution cannot be found. These results obtained after structural optimization at zero temperature are corroborated by AIMD simulations, which confirm the robustness of the closed-shell solution even under the effect of thermal fluctuations at room temperature. Therefore, the *p*-PTM and *p*-BCM appear to be the most effective 1D polymers to induce, respectively, the open-shell multi-radical and closed-shell quinoidal electronic solutions.

Overall, our results demonstrate the powerful role of chemical functionalization to design *p*-TAM polymers with a preferred electronic configuration, and so with desired physicochemical properties. From the structure–property relationship perspective, the ability to tune the dihedral angles of aryl rings appears to be the most effective tool to determine electron pairing or unpairing. We thus believe this study may serve as a general guideline to design 1D polymers with targeted electrical, magnetic, and optical properties based on TAMs or, more generally, π-conjugated organic radicals.

## Author contributions

I. A. and J. C. T. came out with the original idea. I. A. designed the various 1D polymers to be studied and performed all associated simulations. All authors analysed the results and proposed additional calculations. I. A. prepared the first version of the manuscript. All authors contributed to the final paper.

## Conflicts of interest

There are no conflicts to declare.

## Supplementary Material

RA-011-D1RA03187B-s001
