# Supplementary material for: Conformational control over π-conjugated electron pairing in 1D organic polymers
Source: RSC Adv. 2021 Jun 8;11(33):20498–506. doi: 10.1039/d1ra03187b (PMC9033971; doi:10.1039/d1ra03187b)
Supplement: RA-011-D1RA03187B-s001 [file RA-011-D1RA03187B-s001.pdf]

Electronic Supplementary Material (ESI) for RSC Advances.
This journal is © The Royal Society of Chemistry 2021

Electronic Supplementary Information

Conformational control over π-conjugated electron pairing in 1D organic polymers

Isaac Alcón[1]*, Jingjing Shao[1], Jean-Christophe Tremblay[2] and Beate Paulus[1]

[1]*Institut für Chemie und Biochemie, Physikalische und Theoretische Chemie, Freie Universität Berlin, Arnimallee 22, 14195 Berlin, Germany*

[2]*Laboratoire de Physique et Chimie Théoriques, CNRS-Université de Lorraine, UMR 7019, ICPM, 57070 Metz, France*

*Corresponding author: ialcon8@gmail.com

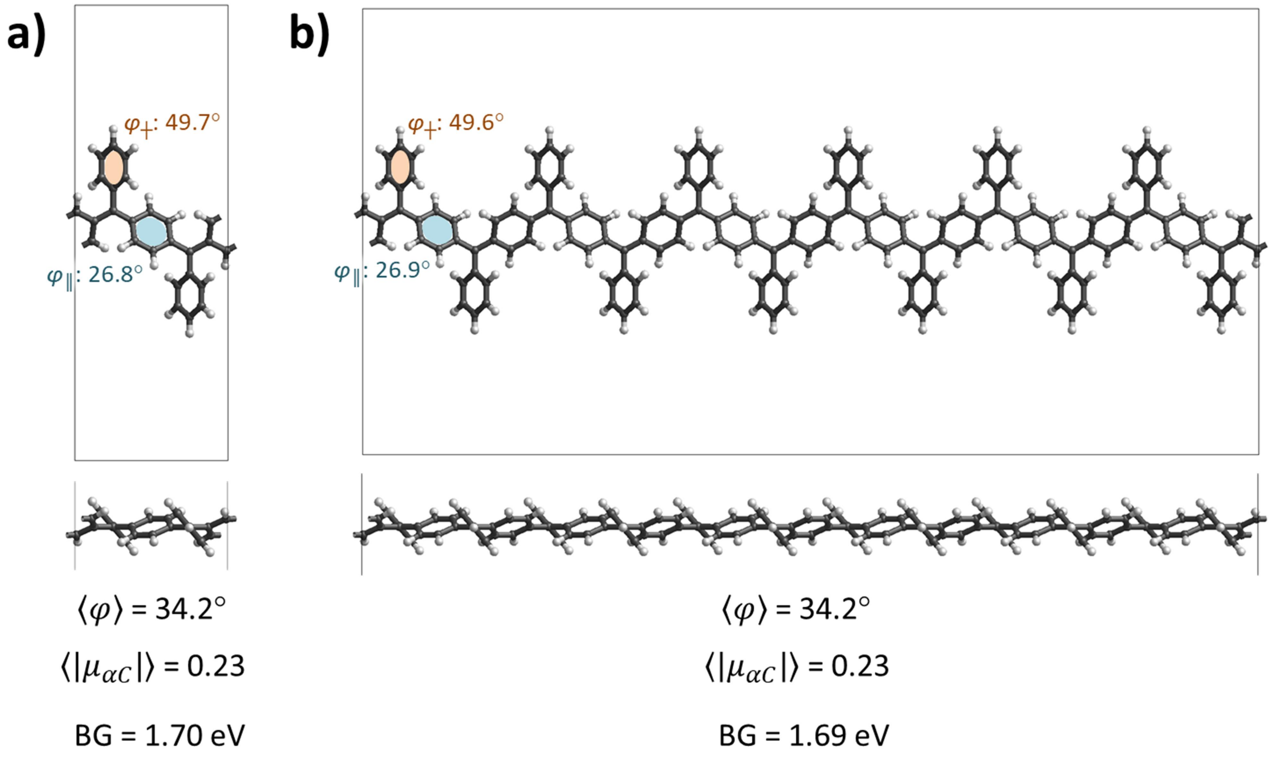

Fig. S1. Comparison of structural and electronic properties for the p-TPM polymer (most flexible considered polymer) obtained upon using **a)** the smallest periodic unit cell utilized throughout our study and **b)** the associated x6 supercell. Both systems where optimized using the settings specified in the Methods section in the main text, except for the smaller k-mesh for the x6 supercell due to its larger size (3 1 1 instead 18 1 1). Some resulting characteristic twist angles for perpendicular ($\varphi_{\perp}$, orange) and parallel aryl rings ($\varphi_{\parallel}$, blue) to the polymeric direction are provided. Key quantities such as the overall mean dihedral angle ($\langle\varphi\rangle$), the average of the absolute αC spin population ($\langle|\mu_{\alpha C}|\rangle$) and the electronic band gap (BG) are also provided for both cases at the bottom. As it may be seen, no apparent new distortions appear for the larger system, leading to almost identical structural and electronic properties for both periodic representations.

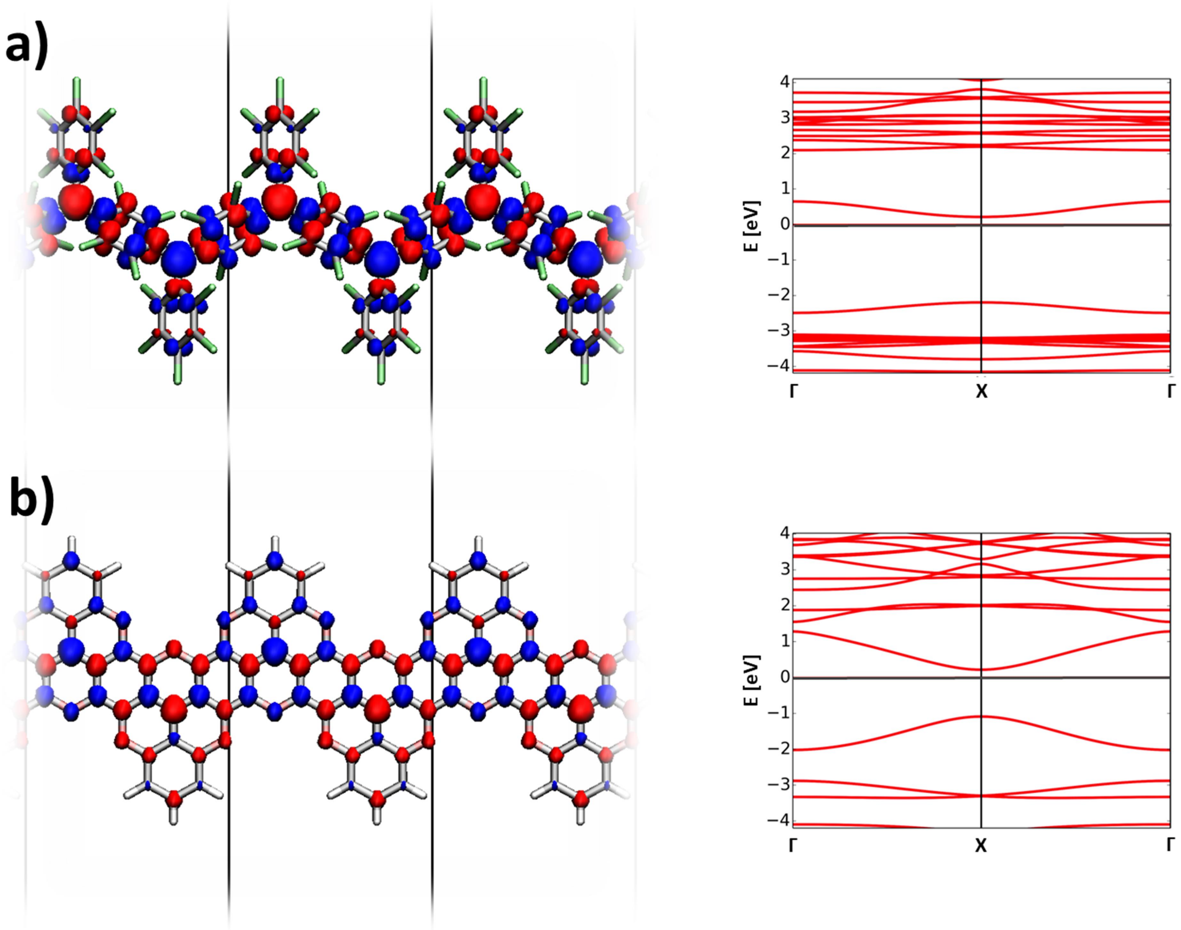

Fig. S2. (left) Spin density isosurface (spin up: blue, spin down: red; iso-surface value: 0.003 e/bohr^3) and (right) associated band structure in the open-shell multi-radical solution for **a)** the p-PTM and **b)** p-oxTAM polymers. Vertical lines indicate the periodic unit cell along the polymer direction. In the band structures the Fermi energy is marked with a black horizontal line.

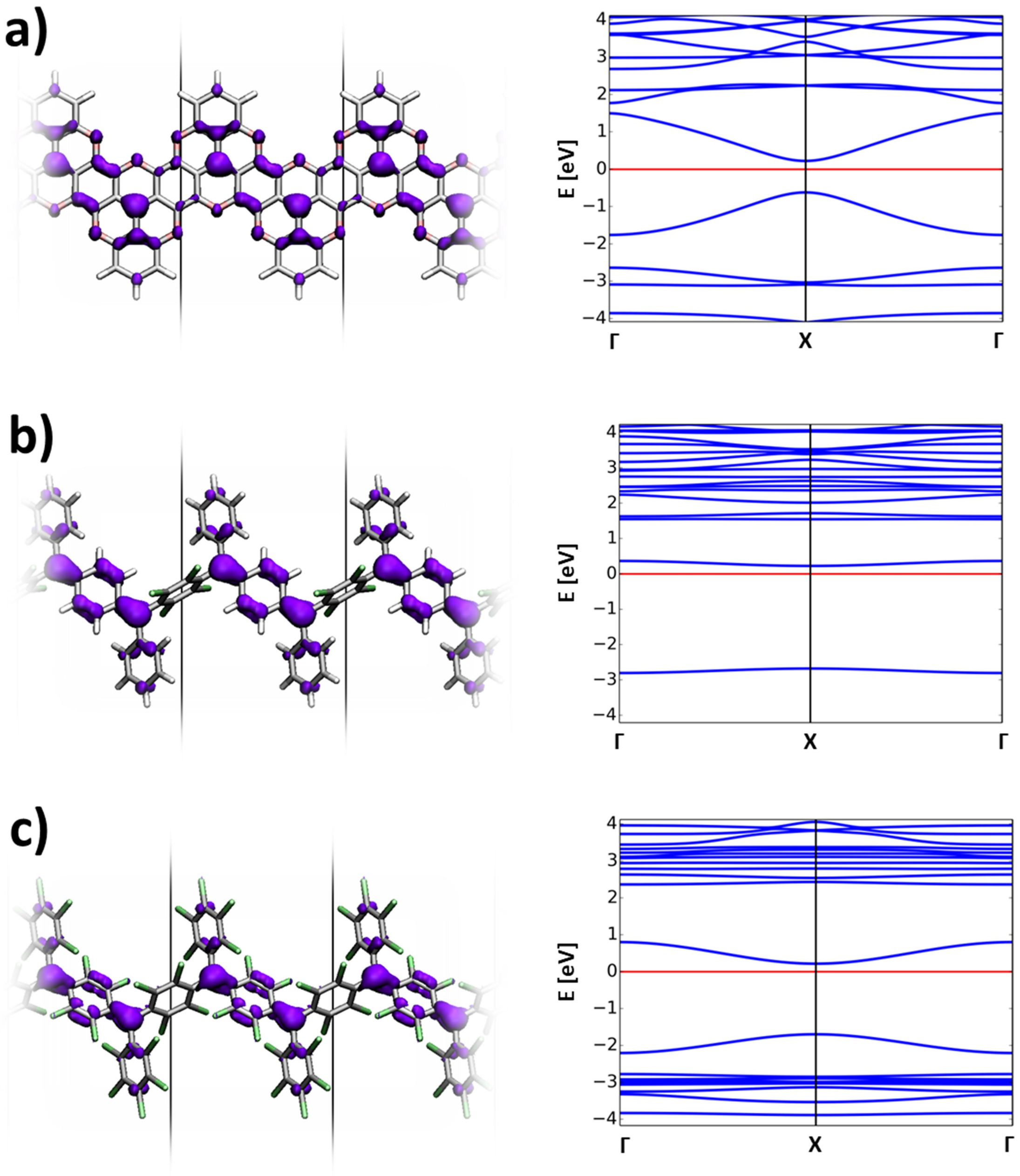

Fig. S3. (left) Highest occupied crystal orbital density (purple; iso-surface value: 0.002 e/bohr^3) and (right) associated band structure in the closed-shell quinoidal solution for **a)** p-oxTAM, **b)** p-BCM and **c)** p-PTM. Vertical lines indicate the periodic unit cell along the polymer direction. In the band structures the Fermi energy is marked with a red horizontal line.

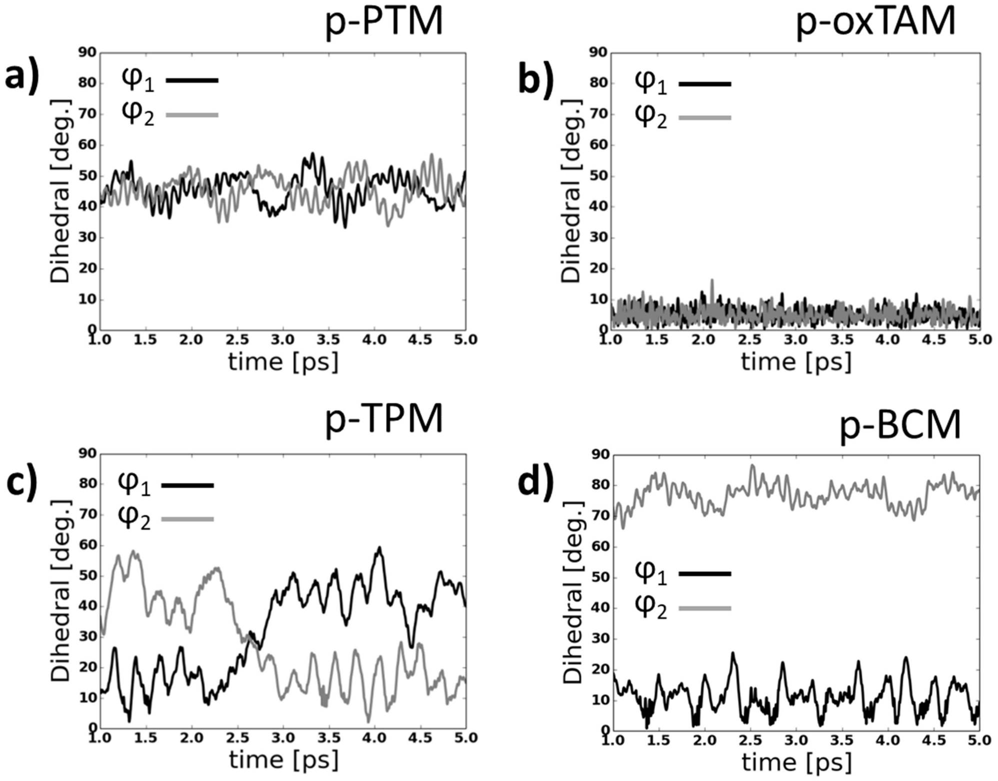

Fig. S4. Variation of the dihedral angles of aryl rings 1 and 2 (φ_1, φ_2 in the main text) during 4 ps of a molecular dynamics at 300 K for **a)** p-PTM, **b)** p-oxTAM, **c)** p-TPM and **d)** p-BCM polymers.

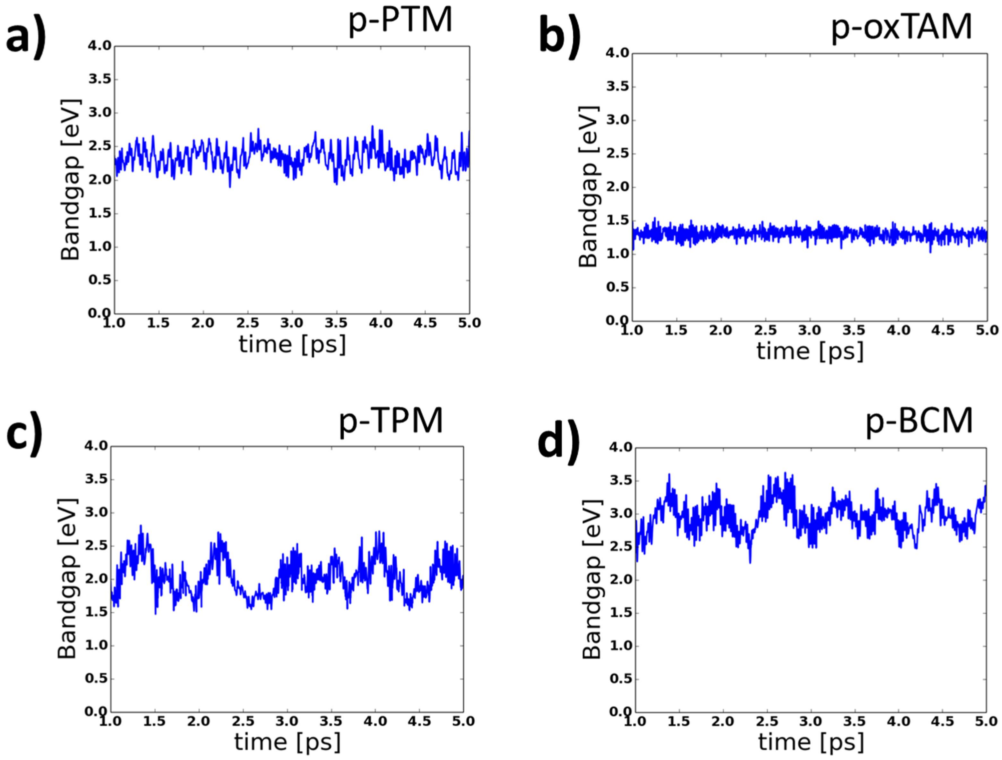

Fig. S5. Variation of the electronic bandgap during 4 ps of a molecular dynamics at 300 K for **a)** p-PTM, **b)** p-oxTAM, **c)** p-TPM and **d)** p-BCM polymers.

Optimized geometries shown in Fig. 2 of the main text (FHI-AIMS format)

Optimized geometry for the p-oxTAM 1D polymer

```
lattice_vector     9.55603583      0.00000000      0.00000000
lattice_vector     0.00000000     30.00000000      0.00000000
lattice_vector     0.00000000      0.00000000     50.00000000
atom       9.53211085     16.43468503     24.98411330 C
atom       9.53820825     17.80375549     24.97022505 O
atom       0.22170052     20.39979956     24.93806621 H
atom       1.19776204     14.28512312     25.00801109 C
atom       1.17405018     15.69287384     24.99259612 C
atom       1.17345344     18.48449365     24.96108979 C
atom       1.16168260     19.86655676     24.94398040 C
atom       2.38150333     13.59956265     25.01597834 O
atom       2.38126906     16.37854234     24.98518994 C
atom       2.38137441     17.79404400     24.96909153 C
atom       2.38152632     20.53759386     24.93470503 C
atom       2.38187188     21.61943158     24.92129605 H
atom       3.58945819     18.48406893     24.95978587 C
atom       3.60106924     19.86620189     24.94207971 C
atom       3.58870712     15.69308569     24.99182325 C
atom       3.56521465     14.28519596     25.00693796 C
atom       4.54112638     20.39930368     24.93489489 H
atom       4.78097615     17.80389400     24.96883872 O
atom       4.78684403     16.43466424     24.98304292 C
atom       4.75411365     13.60212774     25.01366773 C
atom       4.76017115     12.23296192     25.02802404 O
atom       5.97574653     15.75171505     24.99028874 C
atom       5.95217078     14.34379232     25.00547970 C
atom       5.00006543      9.63690568     25.05852901 H
atom       5.95158621     11.55239216     25.03672611 C
atom       5.93994839     10.17030812     25.05276548 C
atom       7.15980534      8.41730613     25.07343302 H
atom       7.15970450      9.49915250     25.06097729 C
atom       7.15951359     12.24275640     25.02912640 C
atom       7.15951271     13.65825132     25.01289519 C
atom       8.37937552     10.17050210     25.05356754 C
atom       8.36754279     11.55255775     25.03700610 C
atom       9.31939308      9.63732271     25.05956961 H
```

atom 0.00282720 12.23302719 25.02815846 O
atom 7.15941714 16.43739430 24.98233340 O
atom 8.36681286 14.34391568 25.00575451 C
atom 8.34313134 15.75174219 24.99058161 C
atom 0.00882915 13.60213952 25.01444390 C

Optimized geometry for the p-TPM 1D polymer

lattice_vector 10.04688270 0.00000000 0.00000000
lattice_vector 0.00000000 30.00000000 0.00000000
lattice_vector 0.00000000 0.00000000 50.00000000
atom 9.94956511 13.82478100 24.90680604 C
atom 9.86597711 12.84680671 24.45067094 H
atom 8.93914881 15.73445667 25.97913674 C
atom 8.78542277 14.43075958 25.44218319 C
atom 8.09402609 16.20529878 26.46338562 H
atom 8.90398587 12.11201001 27.10030882 H
atom 8.89522133 9.64538243 27.13140589 H
atom 8.30800010 11.56233295 26.38211974 C
atom 8.30067423 10.17669286 26.39869076 C
atom 7.53350342 13.74507635 25.44486578 C
atom 7.53384435 12.27116594 25.45708086 C
atom 7.52932418 9.47098296 25.48372413 C
atom 7.52731884 8.38832075 25.49430181 H
atom 6.76078282 10.16141455 24.55488594 C
atom 6.75845013 11.54713419 24.54476285 C
atom 6.16435353 9.61822520 23.83244595 H
atom 6.16408519 12.08487120 23.81624297 H
atom 6.98137060 16.20080006 24.41202457 H
atom 6.28476008 14.42928401 25.43704877 C
atom 6.13393354 15.73270309 24.89477091 C
atom 5.19604654 12.85037516 26.43039097 H
atom 5.11544624 13.82568383 25.96822343 C
atom 4.93087345 16.37967652 24.90145351 C
atom 4.85023439 17.35492879 24.43917536 H
atom 3.88662263 20.58560459 27.03905881 H
atom 3.88441606 18.11901582 27.05430081 H
atom 3.91238074 14.47267143 25.97491053 C
atom 3.76160244 15.77619033 25.43286032 C

atom 3.28911699 20.04332890 26.31682143 C
atom 3.29006917 18.65761145 26.32640263 C
atom 3.06493375 14.00450781 26.45757356 H
atom 2.52384008 21.81757923 25.37856191 H
atom 2.52070713 20.73491605 25.38872385 C
atom 2.51346574 17.93468368 25.41422871 C
atom 2.51300012 16.46076287 25.42555095 C
atom 1.95223170 14.00093408 24.40676203 H
atom 1.74796258 20.03032940 24.47407572 C
atom 1.73932396 18.64467657 24.49006383 C
atom 1.26093614 15.77537867 25.42818261 C
atom 1.10714965 14.47173523 24.89110622 C
atom 1.14236279 18.09586273 23.77202532 H
atom 1.15328037 20.56240498 23.74202660 H
atom 0.18038278 17.35930297 26.41979551 H
atom 0.09678505 16.38136961 25.96356264 C

Optimized geometry for the p-PTM 1D polymer

lattice_vector 10.040241 0.000000 0.000000
lattice_vector 0.000000 30.000000 0.000000
lattice_vector 0.000000 0.000000 50.000000
atom 0.197327 16.063060 25.867894 C
atom 0.457956 17.263469 27.076446 Cl
atom 0.765870 20.870264 22.953852 Cl
atom 0.835925 17.798688 22.863514 Cl
atom 1.022555 14.609788 24.143047 C
atom 1.257110 15.698792 25.010717 C
atom 1.737465 18.615225 24.083311 C
atom 1.728764 20.005785 24.082531 C
atom 2.200068 14.189673 22.955838 Cl
atom 2.515740 16.417247 24.997031 C
atom 2.517546 17.886005 24.994818 C
atom 2.524739 20.704601 24.987986 C
atom 2.529170 22.418856 24.983770 Cl
atom 2.831510 14.200858 27.048900 Cl
atom 3.300960 18.615700 25.903169 C
atom 3.316972 20.006220 25.896968 C
atom 3.773838 15.698148 24.985692 C

atom 4.008555 14.613781 25.858990 C
atom 4.197957 17.801081 27.127562 Cl
atom 4.284695 20.871262 27.021099 Cl
atom 4.572387 17.252481 22.911662 Cl
atom 4.833119 16.058099 24.126099 C
atom 5.217549 13.942637 25.873014 C
atom 5.477848 12.748876 27.088143 Cl
atom 6.042034 15.386810 24.139852 C
atom 6.276980 14.302693 25.013523 C
atom 7.218781 15.799391 22.949572 Cl
atom 5.858410 12.197649 22.871216 Cl
atom 5.768372 9.127753 22.983847 Cl
atom 6.751869 11.384597 24.099258 C
atom 6.734022 9.994161 24.108671 C
atom 7.515183 7.582810 25.030741 Cl
atom 7.522751 9.297059 25.021636 C
atom 7.533992 12.115425 25.007815 C
atom 7.535391 13.584234 25.002969 C
atom 8.317851 9.997023 25.927032 C
atom 8.311646 11.387588 25.922385 C
atom 9.276875 9.133929 27.060008 Cl
atom 9.212386 12.206015 27.141504 Cl
atom 7.850560 15.812612 27.043449 Cl
atom 8.793951 14.302885 24.989247 C
atom 9.028400 15.392051 25.856771 C
atom 9.593821 12.736777 22.924887 Cl
atom 9.853894 13.938269 24.132424 C

Optimized geometry for the p-BCM 1D polymer

lattice_vector 9.82078953 0.00000000 0.00000000
lattice_vector 0.00000000 30.00000000 0.00000000
lattice_vector 0.00000000 0.00000000 50.00000000
atom 0.45327810 15.65130758 26.39131792 C
atom 1.03949056 16.39553057 27.83183954 Cl
atom 0.49886100 20.37989115 23.77422858 H
atom 0.74780413 17.93847311 23.80714936 H
atom 0.75451368 15.08725802 24.08538145 C
atom 1.22759909 15.72637153 25.23344890 C

atom	1.40191876	18.55053339	24.41520236	C
atom	1.26221621	19.92803722	24.39500351	C
atom	1.66881022	15.18569602	22.62457725	Cl
atom	2.50865853	16.47663944	25.22741341	C
atom	2.38937971	17.94059598	25.19702583	C
atom	2.09444890	20.72619359	25.16852931	C
atom	1.98067979	21.80274395	25.15857421	H
atom	2.77885533	13.85627563	25.71648926	H
atom	3.20507794	18.75631724	25.99027175	C
atom	3.06120133	20.13320347	25.97032706	C
atom	3.68722270	15.78117976	25.24403290	C
atom	3.71046103	14.35497968	25.48312362	C
atom	3.92701949	18.29900174	26.65444751	H
atom	3.69584722	20.74565516	26.59857834	H
atom	4.99795058	17.45171298	24.74873806	H
atom	4.96880069	16.40085873	25.00052601	C
atom	4.85271148	13.64497714	25.49062301	C
atom	4.82356842	12.59412494	25.74241946	H
atom	6.11105173	15.69085623	25.00803158	C
atom	6.13428494	14.26465455	25.24710432	C
atom	7.04266045	16.18956433	24.77468440	H
atom	5.89443887	11.74692741	23.83659783	H
atom	6.12559841	9.30026624	23.89231697	H
atom	6.61638606	11.28956342	24.50073377	C
atom	6.76025428	9.91267647	24.52059823	C
atom	7.84079728	8.24308771	25.33221943	H
atom	7.72703550	9.31963896	25.32232508	C
atom	7.43210580	12.10523144	25.29400943	C
atom	7.31284060	13.56918351	25.26368879	C
atom	8.55928375	10.11774524	26.09588643	C
atom	8.41958250	11.49525013	26.07577567	C
atom	9.32266089	9.66585045	26.71660414	H
atom	9.07371716	12.10727590	26.68384120	H
atom	8.15269777	14.86000775	27.86652776	Cl
atom	8.59390073	14.31943414	25.25766026	C
atom	9.06699188	14.95852391	26.40573224	C
atom	8.78198555	13.65024478	22.65929889	Cl
atom	9.36822006	14.39448934	24.09979452	C
